# Preparing Health Systems for Therapeutic Hybridization: Comment on "Routes of Well-Being, Spiritual Harmony and Recovery in Mental Health: The Community as a Policy-Maker"

**DOI:** 10.34172/ijhpm.10065

**Published:** 2026-07-28

**Authors:** Marcela Guapacha-Montoya, Diana Carolina Aguirre-Flórez

**Affiliations:** ^1^Department of Pediatrics, Faculty of Health Sciences, University of Caldas, Manizales, Colombia.; ^2^Faculty of Health Sciences, University of Manizales, Manizales, Colombia.

**Keywords:** Indigenous Peoples, Mental Health Services, Cultural Competency, Community Participation, Health Policy, Colombia

## Abstract

This commentary builds on a policy-oriented understanding of recovery as a co-produced route of well-being, spiritual harmony, and community participation. It examines therapeutic hybridization: the negotiated articulation of biomedical care, ancestral practices, spiritual resources, family support, territorial meanings, and community-based recovery. Rather than treating these arrangements as informal adaptations or cultural supplements, health systems must govern, finance, evaluate, and protect plural forms of care without subordinating Indigenous knowledge. Drawing primarily on Colombian cases and wider literature on community health systems, cultural safety, and intercultural policy, the commentary proposes six domains of preparedness: governance, care pathways, financing, workforce, clinical safety, and learning systems. The agenda is intended as an adaptable, non-exhaustive framework for settings where plural therapeutic systems already interact and where communities can share authority over design and evaluation. Therapeutic hybridization is therefore presented as a test of whether health systems can redistribute legitimacy, resources, and decision-making authority.

 The lead paper makes a relevant contribution to health policy by showing that mental health routes cannot be designed only from clinical, administrative, or epidemiological categories. Mental health is framed as spiritual harmony, collective well-being, safe environments, family support, territorial continuity, identity, and care.^1^ Its strongest contribution is not only methodological, but political: community knowledge becomes a source of policy formulation. This is a substantial shift because many health systems still invite communities to participate while retaining final authority over budgets, indicators, service design, and implementation decisions.

 The next policy question is more difficult: Once recovery is defined through spiritual, territorial, and collective categories, how should health systems respond operationally? A limited answer would be to add cultural adaptation to existing services. A more demanding answer requires preparing health systems for therapeutic hybridization. In this commentary, therapeutic hybridization refers to the negotiated and situated articulation of biomedical care, traditional medicine, spiritual practices, family-based care, community support, and territorial meanings in the organization of recovery. Drawing on Lang’s account of translation and purification, the concept assumes that contact between therapeutic systems does not leave them unchanged: actors translate practices across settings while also redrawing boundaries around what counts as medicine, spirituality, culture, evidence, and risk.^2^

 Therapeutic hybridization is therefore related to, but not interchangeable with, adjacent concepts. Medical pluralism describes the availability and use of multiple therapeutic systems, whether institutions coordinate them. Integrative or intercultural health may create bridges between systems but can remain limited to service coordination or cultural adaptation. Etuaptmumk, or Two-Eyed Seeing, is a co-learning principle for using the strengths of Indigenous and Western ways of knowing together without collapsing one into the other. Therapeutic hybridization adds a narrower health-system question: What institutional transformations are required when these systems interact in actual care? It directs attention to how authority, payment, documentation, referral, safety, and accountability are redistributed at the interface, and to how translation can alter both Indigenous and biomedical practices.^2,3^

 Coexistence allows biomedical and ancestral systems to remain parallel. Hybridization obliges health systems to define points of contact, referral, translation, payment, safety, accountability, and shared authority. In Indigenous mental health, this issue is central. The lead paper and two related Colombian studies conducted by overlapping author teams suggest that cultural practices and belief systems are constitutive of public mental health and that changes in spiritual harmony can occur alongside traditional practices, sanitation, food security, territorial reorganization, and dialogue with biomedical services.^1,4,5^ Because these claims arise from a linked program of research in specific Wayuu and Embera Dobidá settings, they should be read as context-bound and emerging rather than as evidence that the same relationships hold across Indigenous peoples.

 Their value here is to identify mechanisms and health-system questions for testing elsewhere. Families may distinguish between care for the body and care for the spirit while still recognizing that both forms of care can be necessary. The problem, therefore, is not rejection of biomedicine, but the incompleteness of biomedical systems when they cannot recognize spirit, language, territory, collective care, or the legitimacy of traditional therapeutic authority.

 The domains proposed in the Table are not a validated or exhaustive framework. They were derived through a purposive synthesis in two stages. First, the lead paper and related Colombian studies were read for recurrent implementation problems: who decides, how people move across forms of care, who bears costs, what capabilities providers need, how risk is negotiated, and how success is judged. Second, these problems were compared with community health-systems research on place, people, practices, social determinants, public services, informal resources, governance, stewardship, inclusivity, monitoring, ethics, and the interface between community and formal services, as well as with care-pathway work on co-construction, implementation, learning, and scale.^1,4-10^

**Table:** Minimum Preparedness Agenda for Therapeutic Hybridization in Intercultural Mental Health

**Domain**	**Operational Requirement**
Governance	Formal co-governance with Indigenous authorities, traditional healers, families, youth, community representatives, territorial health actors, and decision-makers.
Care pathways	Intercultural referral and counter-referral protocols that include biomedical, ancestral, family, school, and community resources.
Financing	Payment mechanisms for interpretation, transport, traditional care, community follow-up, collective activities, and territorial outreach, not only individual clinical encounters.
Workforce	Mandatory training in cultural safety, anti-racism, structural competency, language mediation, and respectful work with traditional therapeutic authorities.
Clinical safety	Shared protocols for crisis care, medication use, plant–drug interaction review, confidentiality, family accompaniment, and escalation of risk.
Learning systems	Layered indicators combining a small common core of trust, discrimination, continuity, access, and safety with community-defined measures of spiritual harmony, collective recovery, and perceived legitimacy of care.

 The six domains—governance, care pathways, financing, workforce, clinical safety, and learning systems—were retained because each corresponds to a distinct health-system function and failure mode. They are analytically separable but operationally interdependent: governance authorizes decisions; financing makes them feasible; care pathways organize movement; workforce capabilities shape encounters; clinical safety defines acceptable risk; and learning systems provide feedback. Overlaps were therefore treated as expected interfaces, not as reasons to merge domains. The agenda is illustrative and should be adapted or reorganized with communities in different institutional and territorial settings.

 The first domain is governance. Therapeutic hybridization cannot be governed by health professionals alone. Intercultural mental health boards should include legitimate Indigenous authorities, traditional healers, youth, women caregivers, community leaders, primary care teams, mental health professionals, insurers, and territorial decision-makers. Co-governance should not be reduced to consultation; it must include decision-making power over priorities, protocols, budgets, implementation milestones, and evaluation.

 The second domain is care pathways. A hybrid system needs clear routes for movement between family care, traditional care, primary care, specialized mental healthcare, emergency care, and community follow-up. The pathway should not be imagined as a linear referral map. It should function as a living network with multiple entry points: family, school, community leadership, traditional healer, health post, emergency service, or psychosocial team. This is especially important because real help-seeking rarely follows institutional diagrams.

 The third domain is financing. This is probably the most neglected issue. Health systems frequently endorse interculturality while leaving the costs to communities. Interpreters, transport, ceremonial time, traditional healers, family accompaniment, community meetings, and territorial outreach require resources. Without payment mechanisms, hybridization becomes unpaid cultural labour. Colombia’s current implementation of the Indigenous Own and Intercultural Health System creates a policy window for this discussion.^11^ However, the real test will be whether coordination with the general health system includes stable financing, interoperable records, referral rules, workforce support, and recognition of collective forms of care.

 The fourth domain is workforce transformation. Cultural competence is insufficient when it is understood as learning facts about “the other.” Cultural safety requires examining power, racism, institutional routines, and the community’s own assessment of whether care is safe.^12^ Health systems can reproduce epistemic injustice when Indigenous knowledge is discredited or subordinated to Euro-Western frameworks.^13^ In practical terms, training should include local history, Indigenous rights, language mediation, racism in clinical encounters, spiritual explanations of suffering, and negotiation with families and traditional authorities. Training should be evaluated through outcomes, complaints, community feedback, and equity audits, not only attendance certificates.

 The fifth domain is clinical safety. A serious intercultural system must avoid two mistakes: dismissing traditional practices as irrational or romanticizing them as inherently safe. Hybridization requires respectful clinical negotiation. Teams should ask what practices have already been used, what meaning the family attributes to suffering, whether plants or medicines may interact, whether hospitalization prevents essential spiritual care, and what conditions would make biomedical intervention acceptable. Crisis protocols are especially important in suicide risk, psychosis, severe depression, malnutrition, violence, and child protection. Safety should be co-defined, but it cannot remain vague.

 The sixth domain is learning systems. Standard indicators such as service coverage, diagnosis, medication adherence, hospitalization, and symptom change are not enough. A route built around spiritual harmony should also assess trust, discrimination, perceived respect, continuity with traditional care, family support, access to territory-related practices, food and water security, community participation, and whether people feel better in terms that make sense locally. The central measurement problem is to preserve local meaning without making cross-territory learning impossible. A practical solution is a layered indicator set: a small common core of process and safety measures, combined with territory-specific indicators developed through community workshops, narrative methods, and locally anchored rating scales.

 Spiritual harmony, for example, could be assessed through community-generated descriptions of balance and disruption, repeated brief ratings anchored to those descriptions, and qualitative accounts of change. Where pooled quantitative comparisons are intended, item functioning and measurement equivalence should be examined before scores are treated as comparable. Results should be compared across territories at the level of domains and decision rules, not by assuming that identical scores have identical cultural meanings. Indicators should be cognitively tested, reviewed by community governance bodies, and disaggregated where appropriate. Rights-based mental health policy supports a broader view of care, but local indicators must be built with communities rather than imported from global frameworks.^14^

 La Guajira makes this discussion urgent. The region is not only a multicultural context; it is also a territory marked by structural failures in water, food, health access, child nutrition, migration, and Indigenous rights. Current follow-up to the Constitutional Court’s T-302 process shows that responses for Indigenous children in La Guajira still require urgent and long-term measures, including intercultural health actions, genuine dialogue, territorial coverage, and sustainability of interventions.^15^ In this context, therapeutic hybridization cannot be treated as a niche mental health topic. Spiritual harmony is inseparable from water, food, transportation, language, territory, and the ability of Indigenous authorities to participate in health governance.

 There are risks. Formalizing hybrid care can freeze dynamic practices, produce disputes over who represents the community, expose protected knowledge, or turn traditional healers into auxiliary personnel of biomedicine. Hybrid models can also reproduce gendered burdens if women caregivers are expected to sustain continuity without support. These risks are not arguments against hybridization. They are arguments for stronger ethical governance, community ownership of knowledge, and clear safeguards against extraction.

 The proposed agenda should not be transferred as a fixed package. Its relevance beyond Colombia depends on several conditions: more than one therapeutic system is already used in practice; legitimate Indigenous or local authorities can define and revise care arrangements; legal and financing rules permit shared pathways and payment; and communities retain control over protected knowledge and evaluation criteria. Where these conditions are absent, the first task is not scaling a hybrid model but negotiating authority, rights, and safeguards. Transfer should therefore involve local re-derivation of the domains and indicators, not replication of Colombian institutional forms.^9,10,12-14^

 The most important policy implication is not that every setting should adopt a single hybrid model. It is that, where Indigenous and biomedical practices already interact, health systems should stop treating that interaction as administratively invisible. The real question is whether it will remain informal, precarious, and shaped by institutional neglect, or whether it will become part of a just intercultural system. The lead paper shows that communities can become policy-makers. The next step is to ensure that the policies they help create have the legal, financial, clinical, and epistemic infrastructure needed to sustain context-specific therapeutic hybridizations without erasing the worlds of care that make recovery possible.

## Disclosure of artificial intelligence (AI) use

 Not applicable.

## Ethical issues

 Not applicable.

## Conflicts of interest

 Authors declare that they have no conflicts of interest.
